# Fatal gastric volvulus: forensic pathology considerations and postmortem CT findings

**DOI:** 10.1007/s12024-023-00642-1

**Published:** 2023-05-13

**Authors:** Marcus Littlewood, Chris O’Donnell, Sarah Parsons, Melanie S. Archer, Hans H. de Boer

**Affiliations:** 1 University of Queensland Medical School, University of Queensland, 11 Wyndham St, Herston, QLD 4006 Australia; 2 Victorian Institute of Forensic Medicine/Department of Forensic Medicine, Monash University, 65 Kavanagh St, Southbank, Victoria 3006 Australia

**Keywords:** Gastric volvulus, Post-mortem, Autopsy, Cause of death, Forensic radiology, Forensic pathology

## Abstract

Gastric volvulus is a rare cause of gastric obstruction, due to the rotation of the stomach by more than 180°. It is a rare but life-threatening medical emergency that is considered difficult to diagnose at the initial clinical presentation. Forensic pathologists may be presented with gastric volvulus in several ways, for instance, as a cause of sudden and unexpected death or in the context of suspected clinical errors. The post-mortem examination of gastric volvulus may be challenging, due to the specific technical issues it presents and the various mechanisms by which volvulus may cause death. We therefore present five cases of gastric volvulus that in combination represent almost the entire spectrum of presentations and post-mortem findings, to discuss how gastric volvulus may come to the attention of a forensic pathologist, the approach and findings at post-mortem examination (including post-mortem CT), and the variety of mechanisms by which gastric volvulus may result in death.

## Introduction

Gastric volvulus is a rare cause of gastric obstruction, defined by a rotation of the stomach by more than 180°. The malrotation causes acute obstruction of the gastric outlet, inducing severe nausea and vomiting and potential strangulation of the stomach with resultant tissue ischemia and necrosis [1]. Gastric volvulus is a medical emergency when it is acute and requires urgent recognition and management in a clinical setting. Since its mortality is high (up to 42–56%, [2]) and its clinical diagnosis is considered challenging, forensic pathologists are not uncommonly confronted with gastric volvulus, be it as the cause of an unexpected sudden death or in the context of suspected clinical errors.

The lethal potential of gastric volvulus is well-known, but the specific cause and the mechanism of death are variable. In addition, the post-mortem examination of gastric volvulus presents specific technical challenges, although these can be mitigated with proper preparation. There are a few case reports that can aid forensic pathologists when confronted with (suspected) gastric volvulus [3–5], but a comprehensive discussion of its presentation, examination, and interpretation is not available. Therefore, we present five cases of gastric volvulus that in combination represent almost the entire spectrum of presentations and post-mortem findings. We also discuss the various ways in which gastric volvulus may come to the attention of a forensic pathologist, the approach and findings at post-mortem examination (including post-mortem CT; PMCT), and the variety of mechanisms by which gastric volvulus may result in death.

## Epidemiology, classification, and clinical findings

The true incidence of gastric volvulus is unknown, especially since chronic, spontaneously resolving gastric volvulus is underreported. There is no male or female predilection. Adults are by far the most commonly affected, especially after the 5th decade of life. However, about 10–20% occur in children younger than 1 year old [6, 7].

Based on its aetiology, volvulus can be classified as either primary or secondary. Primary volvulus is defined as volvulus due to abnormalities of the gastric ligaments and accounts for approximately a third of all cases [2]. The stomach is anchored by the gastro-oesophageal junction, the pylorus, and the gastrophrenic, gastrosplenic, gastrohepatic, and gastrocolic ligaments. Laxity, agenesis, elongation, and/or disruption of these ligaments can all result in failure to adequately fix the stomach. Secondary gastric volvulus is due to any other cause than a primary ligamentous abnormality. It typically occurs due to diaphragmatic hernias (congenital or acquired) or abdominal adhesions. The first described case of gastric volvulus (by Ambroise Pare in 1579 [8]) was a secondary volvulus with definitive forensic aspects: it was allegedly the direct consequence of a traumatic diaphragmatic hernia from a sword wound. Other rare causes of secondary gastric volvulus include tumours and phrenic nerve palsy [9, 10].

**Fig. 1:**
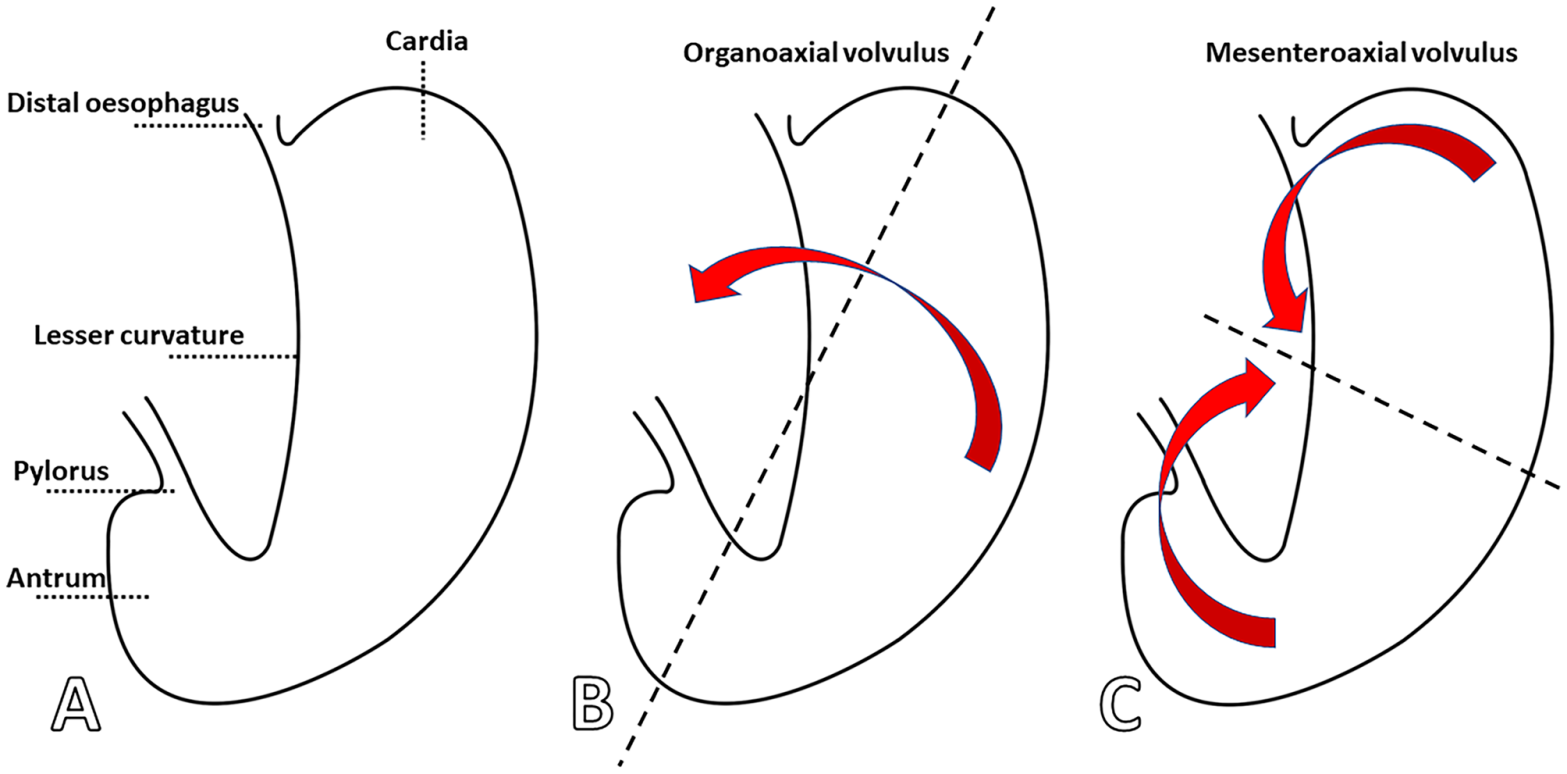
Diagram of the stomach anatomy (**A**), and the rotation in organoaxial (**B**) and mesenteroaxial (**C**) volvulus

Volvulus can also be classified based on the direction of its malrotation (Fig. 1). An organoaxial (OA) volvulus is rotated along its longitudinal axis, i.e. the long axis from the gastric inlet to outlet, and is suggested to resemble “wringing out a wet rag” [6]. A mesenteroaxial (MA) volvulus is rotated along its transverse axis i.e. about an imaginary line from the mid-lesser to the mid-greater curvature. Organoaxial volvulus is approximately twice as common as mesenteroaxial volvulus. In about 10% of cases, a mixed type is observed [2].

The clinical diagnosis of gastric volvulus is considered challenging and requires a high level of suspicion [1]. It should be considered in all patients presenting with acute abdominal pain and symptoms of gastric outlet obstruction. “Borchardt’s triad”, consisting of non-productive vomiting, abdominal pain, and an inability to pass a nasogastric tube, is described in approximately 70% of cases, but a substantial portion of cases present with non-specific findings [7, 11]. In some cases, the gastric volvulus has a chronic component or an intermittent course, with a spontaneous resolution during acute episodes, adding to the diagnostic challenge [12]. Radiological imaging is usually conclusive. Treatment is based on acute decompression through a nasogastric tube, with secondary surgical intervention. The aims of surgery are the reduction of the volvulus and the prevention of recurrence through the repair of predisposing factors [7].

## Case descriptions

### Case 1

A 69-year-old reclusive male was found deceased in his residence after a welfare check.

**Fig. 2:**
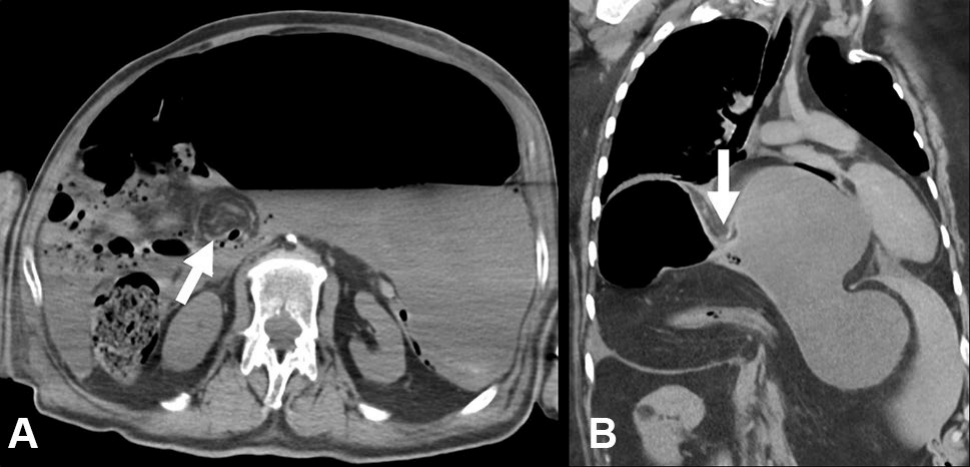
Post-mortem CT findings in gastric volvulus: case 1 (**A**) and case 2 (**B**). Axial reconstruction of the stomach showing massive gas and fluid-filled distension with a lesser omental “whorl” sign (arrow), indicative of organoaxial volvulus. Coronal multiplanar reconstruction showing a distended fluid-filled stomach with “waisting” in the mid gastric body (arrow), indicating the site of organoaxial volvulus within a hiatus hernia

PMCT showed massive distension of the proximal intrathoracic stomach with the distal collapse of the pylorus and duodenum. A “whorl” sign was identified in the lesser omentum indicative of a volvulus (Fig. 2a). The axis of rotation, being perpendicular to the long axis of the gastric body, indicated an organoaxial volvulus.

At the autopsy, moderate decomposition changes were noted. The radiologically observed partially intrathoracic organoaxial gastric volvulus was confirmed, with a markedly distended stomach containing approximately 1500 ml of dark red/brown fluid. There was a sharp mucosal demarcation at the point of torsion, highly suspicious for necrosis, but decomposition hampered its histological confirmation. The gastric herniation caused a mediastinal shift to the left. The lungs were congested and oedematous, but without pneumonic changes on histology. Vitreous humour biochemistry revealed a creatinine of 201 µmol/L and a urea of 29 mmol/L, indicative of moderate renal impairment. Vitreous humour sodium, potassium, and chloride levels were non-diagnostic (114, > 10, and 92 mmol/L, respectively). Toxicological analysis was not contributory.

Death was determined to be caused by gastric volvulus, and the mechanism of death included elements of gastric necrosis, electrolyte disturbances due to vomiting, displacement of the lungs with respiratory compromise, and possible sepsis.

### Case 2

A 68-year-old female with recent complaints of nausea and vomiting, but otherwise no medical history was found deceased at home.

A PMCT scan showed a large hiatus hernia containing a non-dilated transverse colon and a massively distended stomach with a mid-gastric body “waist”, indicative of an organoaxial volvulus (Fig. 2b). There was marked extrinsic compression and displacement of mediastinal structures, including the heart.

**Fig. 3:**
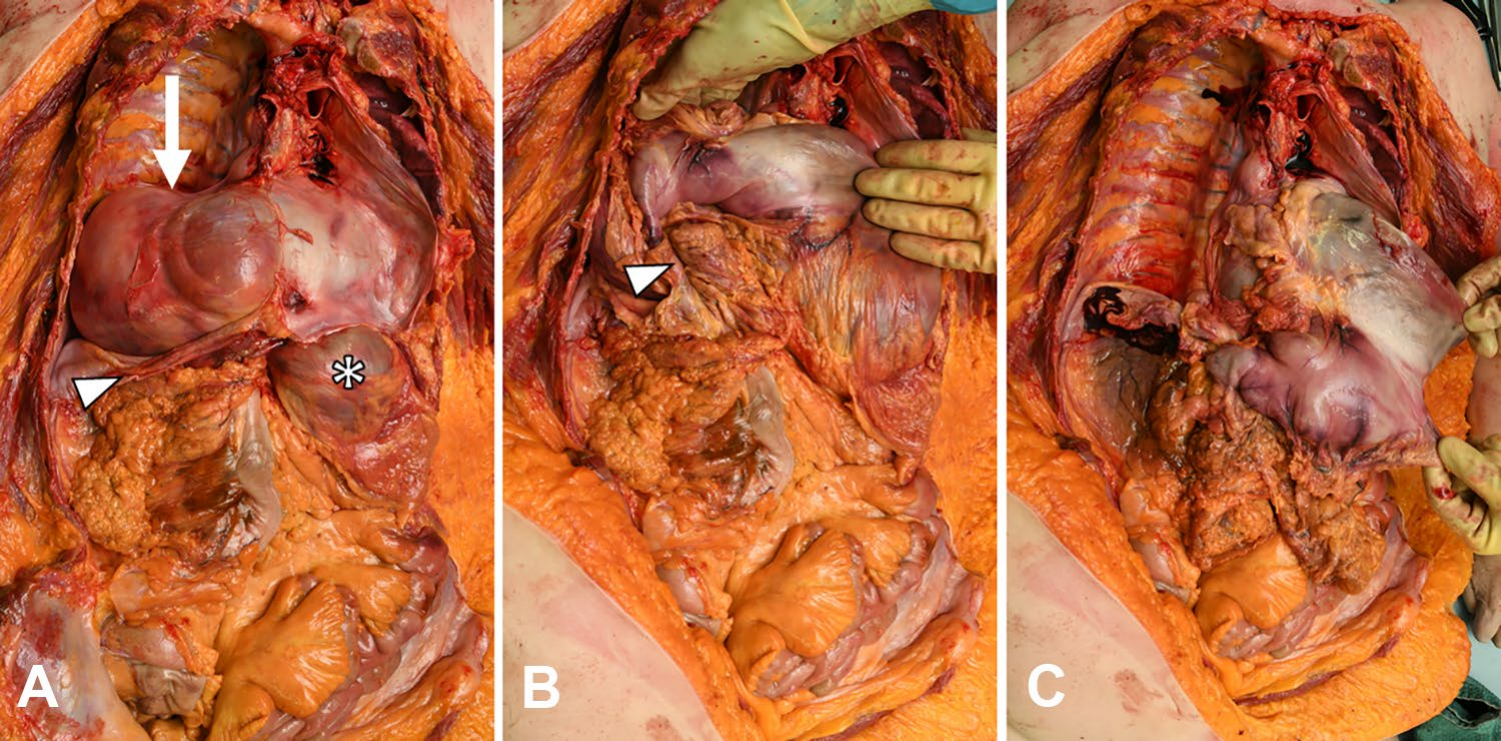
Autopsy findings in a case of intrathoracic organoaxial gastric volvulus (case 2). Removal of the lungs and heart helps to determine the orientation of the stomach, with an intraperitoneal part (asterisk) and an intrathoracic part (arrow), divided by the diaphragm (arrowhead). A loop of transverse colon enters the hernia with the stomach. The diaphragm is opened anteriorly, and the stomach subsequently lifted, showing the volvulus with colonic and omental herniation (arrowhead). The larger curvature of the stomach is retracted inferior-laterally to resolve the volvulus. Note the colour difference of the stomach above and below the level of rotation

The autopsy confirmed the presence of a large hiatus hernia with organoaxial gastric volvulus. The stomach, parts of the greater omentum, and the transverse colon were in the right pleural cavity (Fig. 3). The stomach contained approximately 2500 mL of black, watery fluid and food residue. The gastric mucosa showed irregular, deep brown discolouration but was unremarkable on histological analysis. The lungs showed bilateral acute aspiration pneumonia. Post-mortem biochemistry revealed elevated C-reactive protein (150 mg/L) and normal renal function (vitreous humour creatinine 87 umol/L, urea 7 mmol/L). Vitreous humour electrolytes were within normal post-mortem ranges (sodium 130 mmol/L, potassium > 10 mmol/L, and chloride 103 mmol/L). Toxicological analysis was not contributory.

The cause of death was determined to be complications of gastric volvulus, and the mechanism of death was postulated to include aspiration pneumonia, systemic inflammation, possible electrolyte imbalance due to vomiting, and compression of the thoracic organs.

### Case 3

A 75-year-old female presented to an emergency department from a care facility after several days of recurrent vomiting and abdominal pain. No cause for her complaints was suggested. She was found unresponsive with vomitus on her clothing prior to clinical evaluation, and cardiopulmonary resuscitation was unsuccessful. Her medical history included a hiatus hernia and a previous gastro-intestinal obstruction of unknown cause.

**Fig. 4:**
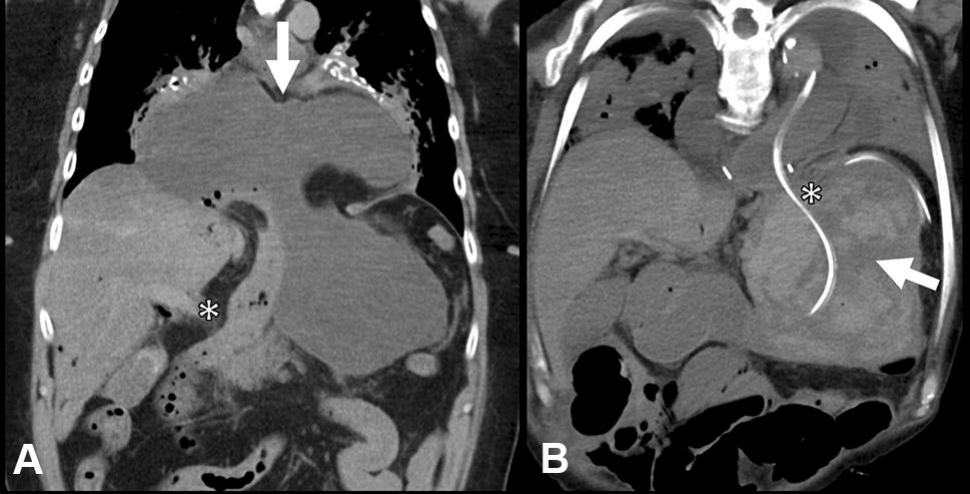
Post-mortem CT findings in gastric volvulus: case 3 (**A**) and case 4 (**B**). Coronal multiplanar reconstruction showing a distended fluid-filled stomach in a hiatus hernia (arrow) with “waisted” mid-gastric body as it passes through the hiatus and extrinsic compression to the pylorus. The proximal duodenum is collapsed (asterisk). Coronal multiplanar reconstruction showing the gastric lumen filled with mixed-density hematoma (arrow). There is no volvulus, due to reduction with a nasogastric tube (asterisk)

PMCT showed a large hiatus hernia containing a distended, fluid-filled stomach with organoaxial volvulus. The distended stomach caused extrinsic compression of the pylorus at the location of the diaphragmatic hiatus (Fig. 4a).

The autopsy confirmed the radiological findings, with a largely intra-thoracic organoaxial gastric volvulus. There was severe dilatation of the oesophagus above the gastro-oesophageal junction and approximately 1100 mL of dark, watery fluid within the stomach. The gastric mucosa was not necrotic. The lungs were consolidated, with abundant gastric contents in the airways and widespread aspiration pneumonia on histology.

The cause of death was determined to be massive aspiration of gastric contents due to gastric volvulus. The mechanism of death probably mainly comprises a combination of aspiration pneumonia and electrolyte imbalance due to vomiting.

### Case 4

A 73-year-old male with a remote history of hiatus hernia repair (Nissen fundoplication) and anterior resection of colon carcinoma presented to an ED with nausea, vomiting, and abdominal pain. He was diagnosed with gastric volvulus on admission. Gastroscopic reduction failed, and he underwent surgical reduction with the repair of a hiatus hernia and the insertion of a percutaneous endoscopic jejunostomy (PEJ) feeding tube. Post-operatively, he developed acidosis and multi-organ failure, with haematemesis and melaena. A re-laparotomy showed gastric mucosal necrosis, perforation of the ascending colon due to ischaemia in the setting of multiorgan failure, and faecal peritonitis. Despite right-sided hemicolectomy and gastric wall oversew, he deteriorated further, was palliated, and died 7 days after admission.

At PMCT, the stomach was distended and filled with heterogenous high-density material, indicative of a haematoma (Fig. 4b). There was a nasogastric tube in situ without signs of residual volvulus, although the oesophagus was still distended.

The autopsy showed gastric mucosal haemorrhage and necrosis with approximately one litre of a blood clot in the stomach. There was a hiatus hernia of 7 cm in the greatest dimension, with the remote Nissen fundoplication confirmed; however, the volvulus was resolved. There was mild peritonitis. The gastric oversew and right-sided hemicolectomy with ileostomy were uncomplicated, but the colon at the splenic flexure showed mucosal ulceration with colonic melaena. Signs of multi-organ failure included pulmonary oedema, pleural effusions, and jaundice with centrilobular hepatic necrosis. The heart was hypertrophic (heart weight 611 g) with previous infarction and severe coronary artery atherosclerosis.

Death was determined to be due to multi-organ failure in the setting of gastric necrosis and peritonitis, following (surgically repaired) gastric volvulus. Pre-existing ischaemic and hypertensive heart disease were listed as contributing factors.

### Case 5

A 68-year-old female with a medical history of hiatus hernia and gastro-oesophageal reflux disease presented to an ED with symptoms of gastrointestinal obstruction. A clinical CT scan was highly suggestive of gastric volvulus, with herniation of almost the entire stomach in the left hemithorax. A diagnostic laparotomy was planned, but she aspirated at anaesthetic induction, and the procedure was abandoned. She developed severe aspiration pneumonia, and despite antibiotic treatment and gastroscopic decompression of the stomach, she deteriorated. She was palliated and died 6 days after admission.

The findings of PMCT included a stomach that was entirely located in the thoracic cavity. There was a nasogastric tube in situ without residual volvulus. A marked thoracic kyphosis was also noted.

The autopsy confirmed a large hiatus hernia with herniation of the stomach and the first 8 cm of the duodenum into the thoracic cavity. There was no volvulus, macroscopic ischemia, or perforation. However, histology showed focal haemorrhage and fat necrosis of the peri-oesophageal and peri-gastric fat, interpreted as evidence of previous strangulation, in keeping with resolved volvulus. Autopsy furthermore confirmed the presence of severe aspiration pneumonia and cardiac hypertrophy (heart weight 533 grammes).

Death was determined to be the result of aspiration pneumonia in the context of gastric obstruction, most likely due to a resolved gastric volvulus.

A summary of the circumstances and relevant post-mortem findings of each case is provided in  Table 1.

**Table 1:** Circumstances and relevant post-mortem findings in five cases of gastric volvulus

**Case**	**Sex, age**	**Circumstances**	**AM diagnosis**	**PMCT findings**	**Autopsy findings**	**Histology findings**	**Ancillary tests**
***1***	M, 69	Reclusive male found deceased at home	N/A	Intrathoracic stomach with OA gastric volvulus and ‘whorl’ sign	Moderate decomposition. Intrathoracic OA volvulus with 1.5 L of altered blood. Sharp demarcation of gastric mucosa at torsion point	Hampered by decomposition	Urea: 29 mmol/L, Creatinine: 201 μmol/L Toxicology not contributory
***2***	F, 68	Found deceased at home. Recent history of nausea and vomiting	N/A	Hiatus hernia with Intrathoracic OA gastric volvulus, omentum and part of the transverse colon. Displacement of thorax organs	Hiatus hernia with OA volvulus, omentum and transverse colon. In the stomach 2.5 L of altered blood and food remnants	Aspiration pneumonia, Gastric mucosa unremarkable	CRP: 150 mg/L Toxicology not contributory
***3***	F, 75	Found deceased in emergency department, 9 h after transfer from rehabilitation clinic for recurrent vomiting	N/A	Hiatus hernia with OA gastric volvulus	Hiatus hernia with partially intra-thoracic OA gastric volvulus containing 1100 mL of altered blood and food remnants. Severely dilated oesophagus. Food residue in the airways	Aspiration pneumonia	Toxicology and biochemistry not requested
***4***	M, 77	Death following large bowel perforation after surgical reduced gastric volvulus	Gastric volvulus complicated by large bowel perforation	Distended stomach containing blood. No residual gastric volvulus	Gastric mucosa haemorrhage/necrosis with 1L bloody stomach contents. Hemicolectomy and gastric perforation repair. Evidence of multi-organ failure. Ischaemic/ hypertensive heart disease	Gastric necrosis	Toxicology and biochemistry not requested
***5***	F, 72	Death after aspiration during anaesthetic induction for diagnostic laparotomy	Gastric obstruction, suspected volvulus	Intrathoracic stomach without volvulus. Thoracic kyphosis	Hiatus hernia with stomach and proximal duodenum in the left thoracic cavity. No volvulus	Aspiration pneumonia, Haemorrhage and fat necrosis of peri-oesophageal and peri-gastric tissues	Toxicology and biochemistry not requested

*AM* ante mortem, *PMCT* post-mortem CT scan, *M* male, *F* female, *N/A* not available, *OA* organoaxial

## Discussion

Our case series illustrates that gastric volvulus can present itself to forensic pathology services in several ways. Although rare, it should be in the differential diagnosis of a sudden and unexpected death at home, especially when scene examination or clinical history indicates recent onset of repetitive and severe vomiting. Due to its challenging clinical diagnosis, presentations also include emergency department admissions, especially when death precedes radiological imaging. A special subset of cases may also be post-procedural or post-operative, in which the diagnosis may be established ante-mortem, although a post-mortem examination should still be considered to detect post-surgical or anaesthetic complications.

Our report is the first to present and summarise the post-mortem radiology findings in a series of gastric volvulus. Overall, PMCT proved to be an important, if not essential, diagnostic adjunct in all our cases. Its major benefit was its ability to readily identify the altered epigastric and thoracic anatomy, thereby enabling the pathologist to plan the dissection. PMCT was also helpful to determine the type of volvulus. Clinical literature indicates that strangulation of the stomach is more common in organoaxial volvulus [1], which would suggest that this type of volvulus is more likely to lead to severe complications. In our series, all volvulus that were still present at post-mortem examination were of the organoaxial type, and these were all associated with sudden and unexpected death.

In line with clinical radiology features [13, 14], gastric volvulus on PMCT is characterised by an abnormally orientated stomach, often but not universally in a hiatus hernia, with evidence of severe gastric obstruction i.e. large volume gastric and sometimes oesophageal dilatation proximally, and a collapsed distal gastric and/or duodenal lumen. The stomach and/or adjacent mesenteric vessels may have a “waisted” or “whorl” appearance. PMCT can also help identify complications, such as perforation and displacement of thoracic organs. However, other essential features such as ischemia of the gastric mucosa or aspiration pneumonia require autopsy and histological confirmation for its diagnosis. Due to the complex altered anatomy, help from a radiologist with experience in post-mortem imaging is advised to interpret the images.

By definition, gastric volvulus implies a complex altered anatomy that may have superimposed surgical treatment. Spontaneous resolution prior to death may complicate matters further. These challenges mandate careful dissection and examination to fully understand the type, severity, and complications of the volvulus. For pre-operative cases, the following recommendations are based on our experience:

If gastric volvulus is suspected prior to the autopsy, re-familiarising oneself with its classification, causes, and complications will greatly reduce confusion during the dissection.

It is not recommended to mobilise organs or perform any dissection before the anatomy is fully appreciated and understood. In particular, deflating the stomach or opening the diaphragm in the anterior midline may spontaneously resolve the volvulus, precluding further examination of its anatomy.

Whether the rotation is organoaxial or mesenteroaxial can be determined relatively easily by following the great curvature of the stomach and tracking any torsion that is present. The greater and lesser omentum are also helpful anatomical landmarks. Separate organs such as the liver, lungs, and heart can be removed for better visualisation, especially when there is a large intra-thoracic component.

Once the anatomy of the volvulus is appreciated, the volvulus can usually be resolved by infero-lateral traction on the greater curvature or omentum. Alternatively, opening the diaphragm in the anterior midline will release a gastric volvulus through a diaphragmatic hernia.

Major complications such as necrosis or perforation of the gastric or colonic mucosa are usually readily recognisable at autopsy. However, histology is essential to confirm complications such as aspiration pneumonia, pancreatitis [15, 16], or necrosis of peri-oesophageal tissues. Fibrosis of the latter may indicate previous strangulation due to chronic volvulus.

In peri- and post-surgical cases, the dissection and interpretation of findings are further complicated by treatment effects and its possible complications. Depending on the extent of surgical intervention, the volvulus may be entirely resolved, and death may be essentially unrelated to the gastric volvulus. Clinical and surgical notes are essential to satisfactorily understand these cases.

Toxicology was requested in two of our cases but was not contributory in either. This supports the notion that, ordinarily, the effects of gastric volvulus explain death without contributing factors. Depending on the context of the case, the exclusion of a toxicological contribution to death may however still be required.

Post-mortem biochemistry is generally considered difficult to interpret, but elevated levels of C-reactive protein (CRP), sodium, chloride, urea, and creatinine may help to examine the respective contributions of systemic inflammation, dehydration, vomiting, and renal impairment to the mechanism of death. For instance, in our first two cases, both presenting as sudden and unexpected death, the biochemical results suggested contributions of renal impairment and systemic inflammation. In peri-operative deaths, suspected anaphylaxis may prompt testing for tryptase.

Literature suggests that gastric necrosis and ischemia are the main complications of gastric volvulus [1, 3, 17], but in our series, aspiration pneumonia was an important mechanism by which death occurred. In all our cases that presented as unexpected death at home, severe dilatation of the proximal stomach was noted, furthermore illustrating the risk of massive and lethal aspiration that gastric volvulus carries. Ischemic necrosis of the gastric mucosa was noted in two of our cases, with one further complicated by perforation, peritonitis, and massive mucosal haemorrhage.

A less commonly known complication of gastric volvulus is the displacement of intrathoracic organs, possibly limiting respiration and cardiac output. The known relation between kyphoscoliosis and gastric volvulus is especially relevant in this regard, since these individuals carry a higher risk of gastric volvulus, whilst having a more restricted thoracic cavity [5, 7, 18]. In three of our cases, a substantial intrathoracic portion of the volvulus was noted, with the potential to substantially compromise (cardio)respiratory function. One of these had kyphoscoliosis. Our second case illustrates that the mass effect of the distended, fluid-filled stomach may be compounded by the intra-thoracic presence of other structures, such as the mesentery or colon. Fluid shifts and electrolyte disturbances are more difficult to appreciate at post-mortem but are mentioned as potential complications in a clinical context [1]. Especially in cases of severe vomiting and/or rapid intermittent volvulus, dehydration and electrolyte disturbances are hypothetically relevant.

Our fourth case illustrates that the abovementioned complications may also extend into the post-operative period. A similar case was described by Sleiwah et al. [17]. Peri- and post-operative cases can also include a variety of surgical and anaesthetic complications. In our series, only aspiration at anaesthetic induction was noted, but other complications should nonetheless be considered in all peri- and post-operative deaths. Such complications include anastomosis leaks, vascular injury, ischaemia, infection, drug effects, and anaphylaxis.

## Key points

1. Gastric volvulus is a rare but life-threatening type of gastric obstruction that may present to a forensic pathologist in many ways.
2. Five cases of gastric volvulus are presented, to discuss its presentation, its post-mortem findings, and the various mechanisms of death.
3. Post-mortem CT is an extremely valuable adjunct in the investigation of gastric volvulus.
4. Practical recommendations are provided to assist in the careful dissection required to determine the type of gastric volvulus and to examine for complications.
5. The mechanisms of death may vary per case and may include aspiration, gastric necrosis, peritonitis, cardiorespiratory compromise due to mass effect, and peri- or post-operative complications.

## Data Availability

The data that support the findings of this study can be requested from the corresponding author [HHdB]. Legal and ethical restrictions may apply.
